# A Case of Minimal Change Disease in a Patient Receiving Immune Checkpoint Inhibitor

**DOI:** 10.7759/cureus.115185

**Published:** 2026-08-25

**Authors:** Magnus Hanbin Liew, Anil Xavier, Andrea Chui Rong Chieng

**Affiliations:** 1 Internal Medicine, Goulburn Valley Health, Shepparton, AUS; 2 General Medicine/Nephrology, Goulburn Valley Health, Shepparton, AUS

**Keywords:** immune-checkpoint inhibitor adverse effects, melanoma and immunotherapy, minimal change disease (mcd), nephrotic-range proteinuria, nephrotic syndorme

## Abstract

Immunotherapies, particularly immune checkpoint inhibitors, are increasingly used in cancer treatment but can cause immune-related adverse events, including rare renal complications. We report the case of a 72-year-old man with metastatic melanoma receiving combination ipilimumab and nivolumab who presented with progressive bilateral lower limb oedema. Investigations demonstrated severe hypoalbuminemia and nephrotic-range proteinuria. Autoimmune investigations were unremarkable, while kidney biopsy showed diffuse podocyte foot process effacement without immune deposits on electron microscopy, consistent with minimal change disease. The patient was treated with high-dose prednisolone, diuretics and albumin replacement, resulting in resolution of oedema, recovery of serum albumin and progressive reduction in proteinuria. Immune checkpoint inhibitor-associated minimal change disease is believed to be mediated by dysregulated cytotoxic T-cell activation causing podocyte injury. Non-steroidal anti-inflammatory drug exposure may potentially increase susceptibility to podocyte injury and the development of minimal change disease. This case highlights the importance of recognising nephrotic syndrome as a rare immune-related adverse event of immune checkpoint inhibitor therapy, as prompt diagnosis and corticosteroid treatment are associated with favourable renal outcomes.

## Introduction

Immune checkpoint inhibitors are an established treatment modality for a wide range of solid and haematological malignancies due to their demonstrated efficacy and survival benefit [1]. However, their use is associated with immune-related adverse events affecting multiple organ systems. The most frequently reported immune-related adverse events include colitis, hepatitis, dermatitis and hypophysitis [2]. Renal immune-related adverse events are uncommon but clinically important, as delayed recognition may result in misdiagnosis, inappropriate management and adverse renal outcomes. As the use of immune checkpoint inhibitors continues to expand, the incidence of renal immune-related adverse events is expected to increase.

Renal immune-related adverse events are rare and most frequently manifest as acute kidney injury secondary to acute tubulointerstitial nephritis [3]. Approximately 5% of patients receiving combination cytotoxic T lymphocyte antigen 4 (CTLA-4) and programmed cell death protein 1 (PD-1) inhibitor therapy develop acute tubulointerstitial nephritis [3]. In contrast, minimal change disease associated with immune checkpoint inhibitors is rarely reported. A systematic review by Seshadri et al. [4] identified only 20 cases of biopsy-proven immune checkpoint inhibitor-associated minimal change disease. Pembrolizumab, a PD-1 inhibitor, was implicated in the majority of cases (50%), followed by nivolumab (25%) and ipilimumab (25%). Encouragingly, most patients demonstrated complete remission of proteinuria following corticosteroid therapy.

We report the case of a 72-year-old man with metastatic melanoma receiving combination ipilimumab and nivolumab who presented with progressive bilateral lower limb oedema. Comprehensive evaluation, including electron microscopy of a kidney biopsy, confirmed minimal change disease in a patient receiving immune checkpoint inhibitor therapy. The patient was treated with high-dose corticosteroids, diuretics and albumin replacement, resulting in progressive reduction in proteinuria, recovery of serum albumin levels and resolution of oedema.

## Case presentation

A 72-year-old man with a background of metastatic melanoma presented to the oncology unit for his third cycle of combination immunotherapy with ipilimumab and nivolumab. On review, he reported progressive bilateral lower limb swelling over the preceding month. He denied associated symptoms, including lower limb pain, shortness of breath, orthopnoea, paroxysmal nocturnal dyspnoea, chest pain or recent infections. His distant past medical history was otherwise unremarkable, with no significant comorbidities besides hypertension. He denied any family history of cardiac, liver or kidney disease. However, he had been admitted a month earlier with acute kidney injury, detected on a routine blood test by his general practitioner (GP). His estimated glomerular filtration rate (eGFR) had dropped from a baseline above 90 to 20, prompting the GP to refer him to the emergency department for further investigation. On further history, he had been taking ibuprofen 400 mg three times daily for the past two weeks for pain at his melanoma site, in addition to his regular valsartan for hypertension. Other investigations were unremarkable, with normal electrolyte and albumin levels, and no significant proteinuria or albuminuria. He was treated for prerenal acute kidney injury with intravenous fluids and by withholding valsartan and stopping ibuprofen. He responded well to hydration and non-steroidal anti-inflammatory drug (NSAID) avoidance, with full recovery of his kidney function.

**Figure 1:**
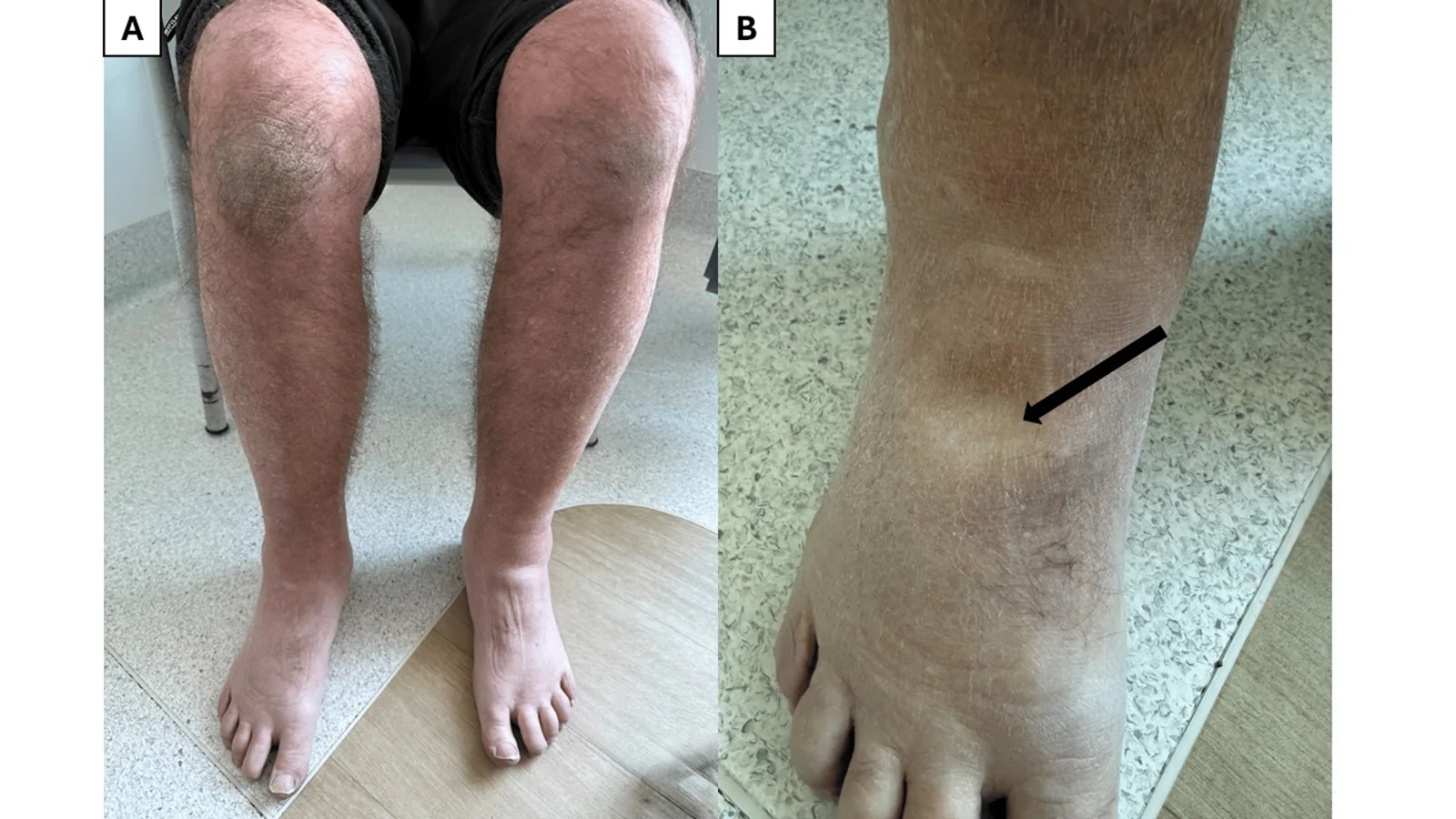
Clinical finding (A) Bilateral lower limb and pedal oedema. (B) Right pedal pitting oedema, demonstrated by indentation of the swelling on palpation (black arrow).

On examination, there was extensive bilateral lower limb pitting oedema extending all the way to his sacrum (Figure 1). Cardiopulmonary examination was unremarkable, and on abdominal examination, there was no hepatosplenomegaly. Laboratory investigations revealed profound hypoalbuminemia, with a serum albumin level of 14 g/L. He was subsequently admitted for further evaluation and management of oedema and severe hypoalbuminemia. 

Urinalysis demonstrated marked proteinuria, with a urine protein-to-creatinine ratio of 1385.89 mg/mmol and urine albumin-to-creatinine ratio of 940.4 mg/mmol, which equated to about 13-14 g/day of proteinuria. Urine microscopy and culture were unremarkable, with no casts, erythrocytes or evidence of infection. An extensive autoimmune workup, including antinuclear antibodies (ANA), anti-double-stranded DNA antibodies (anti-dsDNA), complement levels (C3 and C4), antineutrophil cytoplasmic antibodies (ANCA) and anti-glomerular basement membrane (anti-GBM) antibodies, was negative.

Blood tests revealed elevated globulin levels, prompting screening for underlying multiple myeloma, as it can uncommonly cause minimal change disease. Serum protein electrophoresis demonstrated an elevated gamma globulin of 22 g/L, with mildly elevated alpha-1 globulin of 6 g/L and alpha-2 globulin of 10 g/L. Serum free light-chain analysis showed elevated kappa free light chain at 56 mg/L and lambda free light chain at 55 mg/L, with a normal kappa/lambda ratio of 1.02. These findings were consistent with polyclonal hypergammaglobulinemia, without evidence of a monoclonal gammopathy. The proportionate elevation of both serum free light chains, with a preserved kappa/lambda ratio, together with the absence of a monoclonal band on serum protein electrophoresis, made a monoclonal plasma cell disorder such as multiple myeloma unlikely as the cause of the elevated globulin level. The polyclonal hypergammaglobulinemia may instead reflect chronic immune activation associated with metastatic melanoma and potentially immune stimulation from immune checkpoint inhibitor therapy. The investigation results are summarised in Table 1.

**Table 1:** Summary of investigation results Investigation results demonstrated severe hypoalbuminemia and nephrotic-range proteinuria. eGFR: estimated glomerular filtration rate; ALT: alanine aminotransferase; AST: aspartate aminotransferase; GGT: gamma-glutamyl transferase; ALP: alkaline phosphatase; ANA: anti-nuclear antibody; dsDNA: double-stranded DNA; ANCA: antineutrophil cytoplasmic antibodies; GBM: glomerular basement membrane; C3: complement component 3; C4: complement component 4; K/L ratio: kappa/lambda ratio; MCS: microscopy, culture, and sensitivity.

Test	Results	Units	Reference range
Haemoglobin	135	g/L	130-170
White cell count	8.1	x10^9^/L	4.0-12.0
Platelet	357	x10^9^/L	150-400
Neutrophil	4.3	x10^9^/L	2.0-8.0
Sodium	135	mmol/L	135-145
Potassium	4.1	mmol/L	3.5-5.2
Chloride	98	mmol/L	95-110
Bicarbonate	27	mmol/L	22-32
Urea	8.1	mmol/L	3.2-7.4
Creatinine	82	umol/L	60-110
eGFR	83	-	>90
Total protein	61	g/L	60-80
Albumin	14	g/L	35-50
Globulin	46	g/L	25-40
Bilirubin	7.5	umol/L	<20.0
ALT	40	U/L	6-40
AST	53	U/L	6-35
GGT	29	U/L	5-50
ALP	158	U/L	30-110
ANA	Negative	-	-
dsDNA	<9.8	IU/ml	Negative <27 IU/ml
ANCA	Negative	-	-
GBM antibody	<3	CU	<20
C3	1.50	g/L	0.82-1.85
C4	0.42	g/L	0.15-0.53
Alpha 1 globulin	6	g/L	2.1-3.5
Alpha 2 globulin	10	g/L	5.1-8.5
Beta 1 globulin	4	g/L	3.4-5.2
Beta 2 globulin	4	g/L	2.3-4.7
Gamma globulin	22	g/L	8.0-13.5
Kappa free light chain	56	mg/L	3.3-19.4
Lambda free light chain	55	mg/L	5.7-26.3
K/L ratio	1.02	-	0.26-1.65
Urine MCS	No growth, no red cell cast	-	-
Urine albumin: creatinine ratio	940.4	mg/mmol	<2.5
Urine protein: creatinine ratio	1385.89	mg/mmol	<30.00

A provisional diagnosis of nephrotic syndrome was made, and a kidney biopsy was arranged. The patient was treated with diuretics, albumin replacement and high-dose prednisolone following the biopsy. Light microscopy of the kidney biopsy specimen was unremarkable, with no crescents, segmental sclerosing lesions or necrosis. There was no inflammation or infiltrate of immune cells to suggest interstitial nephritis. Electron microscopy showed extensive podocyte foot process effacement with microvillous transformation. No immune deposits or other significant morphological abnormalities were identified. A diagnosis of minimal change disease was made in a patient receiving immune checkpoint inhibitor therapy. The oncology team withheld ipilimumab and nivolumab, given their suspicion that the immune checkpoint inhibitor therapy was the likely cause of the minimal change disease, which had resulted in severe hypoalbuminemia, peripheral oedema, and nephrotic-range proteinuria.

**Figure 2:**
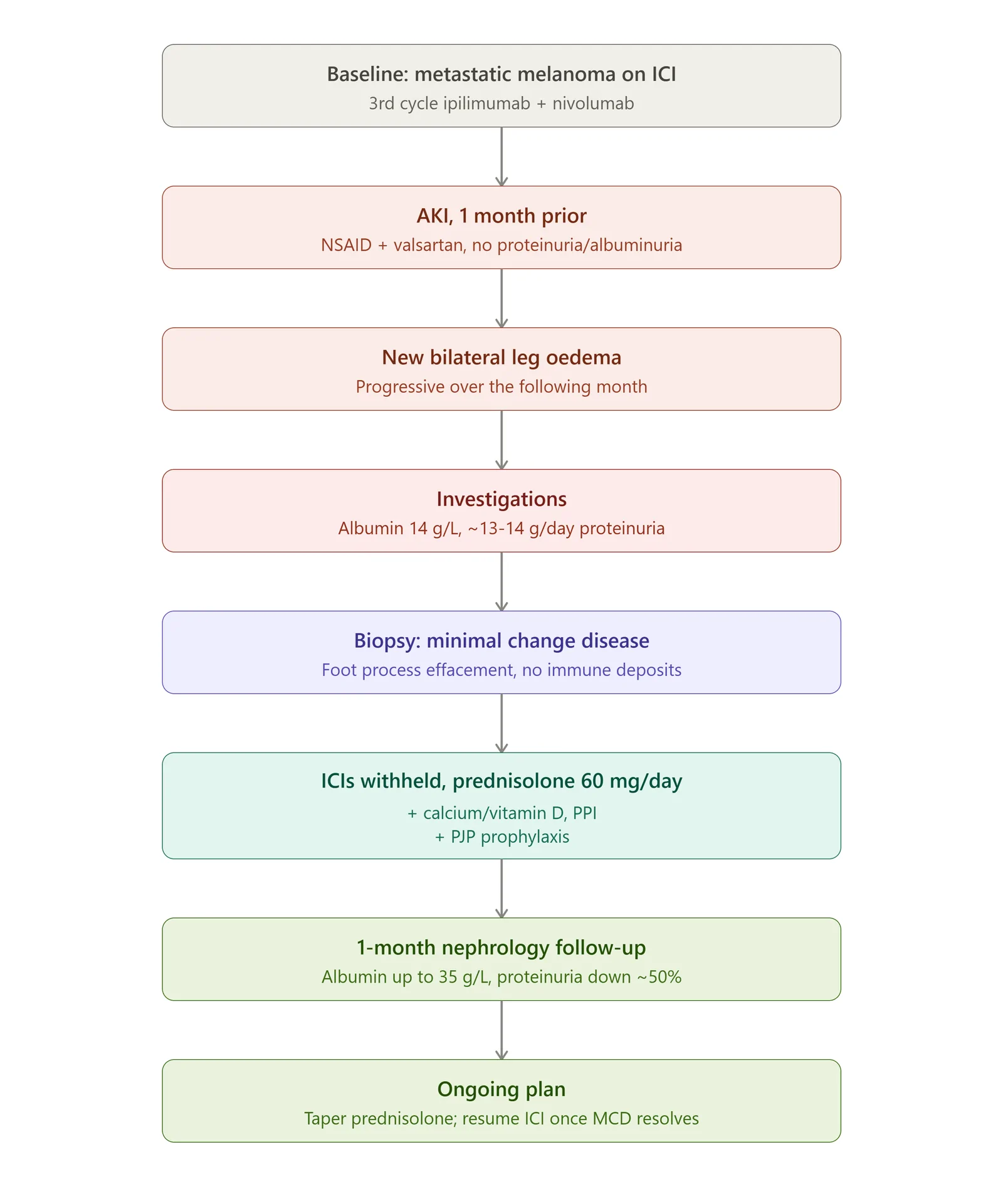
Clinical timeline Clinical timeline summarising events from presentation with bilateral lower limb oedema, through investigations revealing hypoalbuminemia and nephrotic-range proteinuria, to biopsy-proven minimal change disease and subsequent recovery on prednisolone therapy. ICI: immune checkpoint inhibitor; AKI: acute kidney injury; NSAID: non-steroidal anti-inflammatory drug; PPI: proton pump inhibitor; PJP: *Pneumocystis jirovecii *pneumonia; MCD: minimal change disease

The patient was commenced on high-dose prednisolone at 60 mg once daily, together with prophylactic medications including calcium and vitamin D supplementation, pantoprazole and trimethoprim-sulfamethoxazole for *Pneumocystis jirovecii *pneumonia (PJP) prophylaxis. He was subsequently discharged with ongoing nephrology follow-up. He was reviewed in the nephrology clinic after one month, with repeat blood and urine investigations. His serum albumin had increased to 35 g/L, while his urine albumin-to-creatinine ratio had decreased by approximately 50% to 435 mg/mmol, indicating a favourable response to corticosteroid therapy. The prednisolone dose was planned to be tapered by 10 mg every four weeks, with monthly blood and urine monitoring to assess the ongoing response, including serum albumin and urine albumin-to-creatinine ratio. As the patient's proteinuria decreased and serum albumin levels improved following prednisolone therapy, the oncology team planned to resume ipilimumab and nivolumab once the minimal change disease had completely resolved. The patient's clinical timeline is summarised in Figure 2.

## Discussion

Ipilimumab is a cytotoxic T lymphocyte antigen 4 (CTLA-4) inhibitor, while nivolumab is a programmed cell death protein 1 (PD-1) inhibitor [1]. These agents are immune checkpoint inhibitors, which enhance antitumor immunity by blocking inhibitory signalling pathways that normally downregulate cytotoxic T-cell activation, thereby promoting the recognition and destruction of malignant cells. However, this heightened immune activation can impair self-tolerance and promote autoimmunity, resulting in immune-related adverse events.

Current literature supports an association between minimal change disease and multiple classes of immune checkpoint inhibitors, postulating a shared immunological mechanism underlying this form of renal pathology. However, the precise pathophysiology of immune checkpoint inhibitor-associated minimal change disease remains incompletely understood. It has been proposed that dysregulation and upregulation of autoreactive cytotoxic T cells may lead to podocyte injury, resulting in foot process effacement [5]. This pathophysiological cascade manifests clinically as heavy proteinuria, hypoalbuminemia, and nephrotic syndrome, as seen in our patient.

Interestingly, NSAID use has been associated with nephrotic syndrome, most commonly manifesting as minimal change disease on kidney biopsy [6]. NSAID-associated minimal change disease is often characterised by concurrent features of acute interstitial nephritis and diffuse podocyte foot process effacement [6]. The underlying pathogenesis is thought to involve a delayed-type hypersensitivity reaction, whereby NSAID exposure activates T lymphocytes and promotes the release of cytokines that injure podocytes [7]. In addition, cyclooxygenase inhibition by NSAIDs reduces prostaglandin synthesis, leading to increased shunting of arachidonic acid metabolism toward leukotriene production [7]. This process may further activate T-helper cells and contribute to podocyte injury. Reduced prostaglandin levels also impair renal perfusion, thereby exacerbating renal injury and disease progression [7]. Although our patient's kidney biopsy did not show features of acute interstitial nephritis, NSAID-induced minimal change disease cannot be entirely excluded, as it is more common and can present with diffuse podocyte foot process effacement alone. However, the absence of hypoalbuminemia and proteinuria during his earlier admission for NSAID-induced acute kidney injury suggests that NSAIDs were less likely to be the major cause of his minimal change disease. Given his exposure to both NSAIDs and immune checkpoint inhibitors, these factors may have acted synergistically to increase his susceptibility to developing minimal change disease.

## Conclusions

With the increasing use of immune checkpoint inhibitors in oncology practice, greater emphasis is required on recognising renal immune-related adverse events. Although rare, minimal change disease is a clinically important entity, given its generally favourable response to corticosteroid therapy. Future research should aim to further elucidate the underlying immune mechanism driving immune checkpoint inhibitor-associated minimal change disease and to define optimal long-term management strategies, including the safety of rechallenge with immune checkpoint inhibitors and the role of concurrent immunomodulatory therapies.
